# Supplementary figures and images for: Identification and Validation of the Prognostic Panel in Clear Cell Renal Cell Carcinoma Based on Resting Mast Cells for Prediction of Distant Metastasis and Immunotherapy Response
Source: Cells. 2023 Jan 1;12(1):180. doi: 10.3390/cells12010180 (PMC9818872; doi:10.3390/cells12010180)

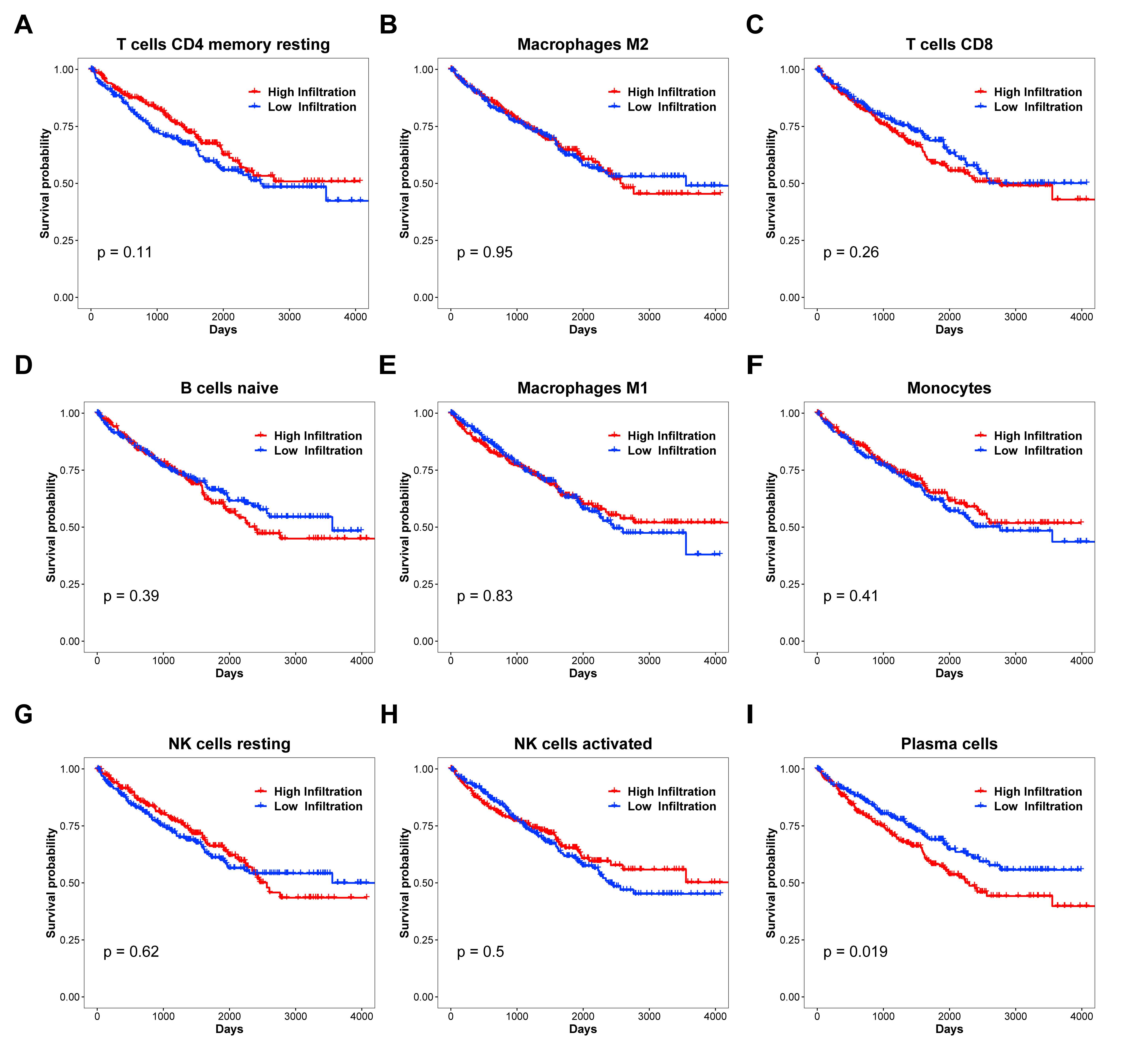

Supplement: Supplementary file 1 [file cells-12-00180-s001.zip › Supplementary Figures/FIgure S1.jpg]

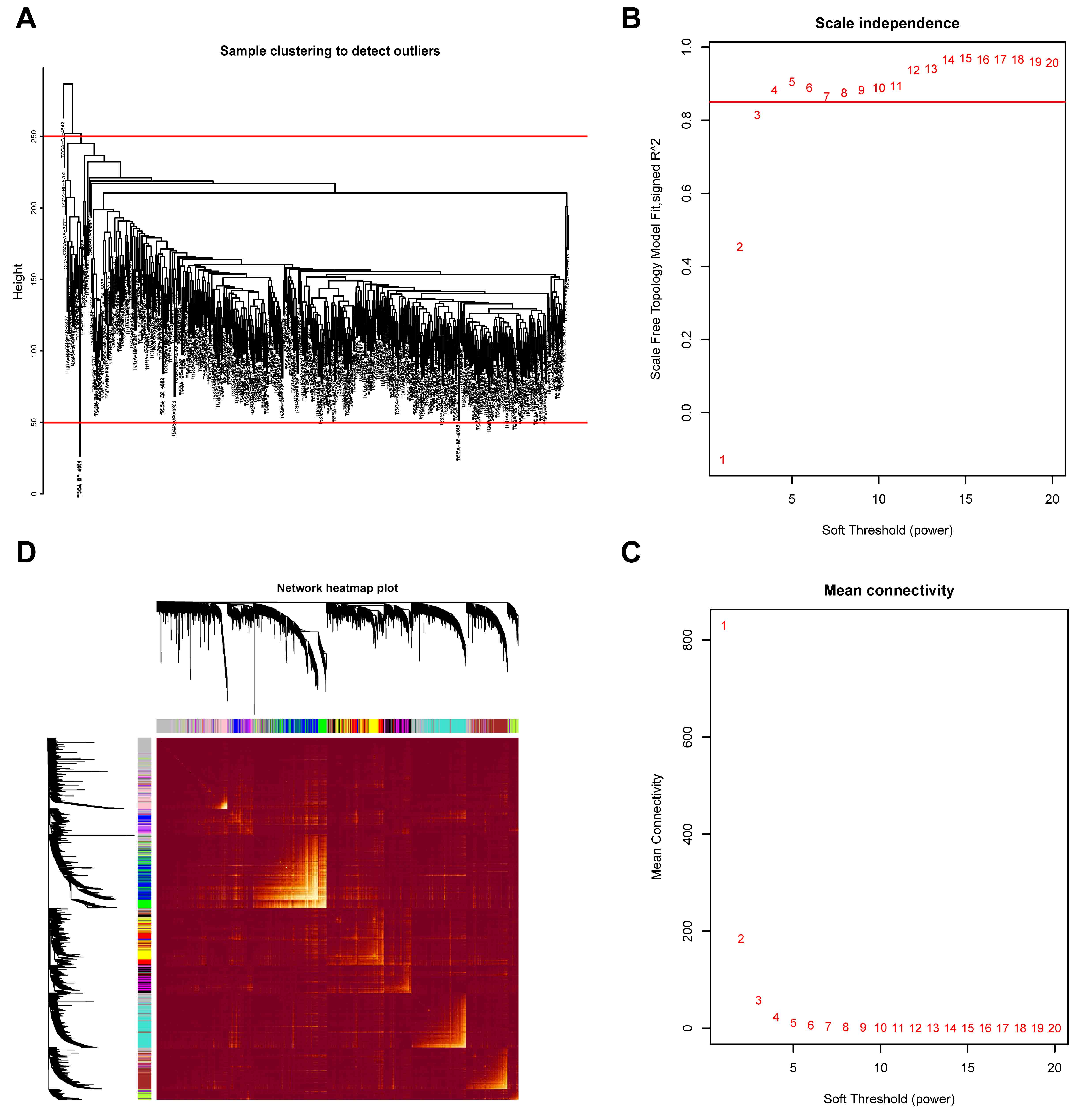

Supplement: Supplementary file 1 [file cells-12-00180-s001.zip › Supplementary Figures/FIgure S2.jpg]

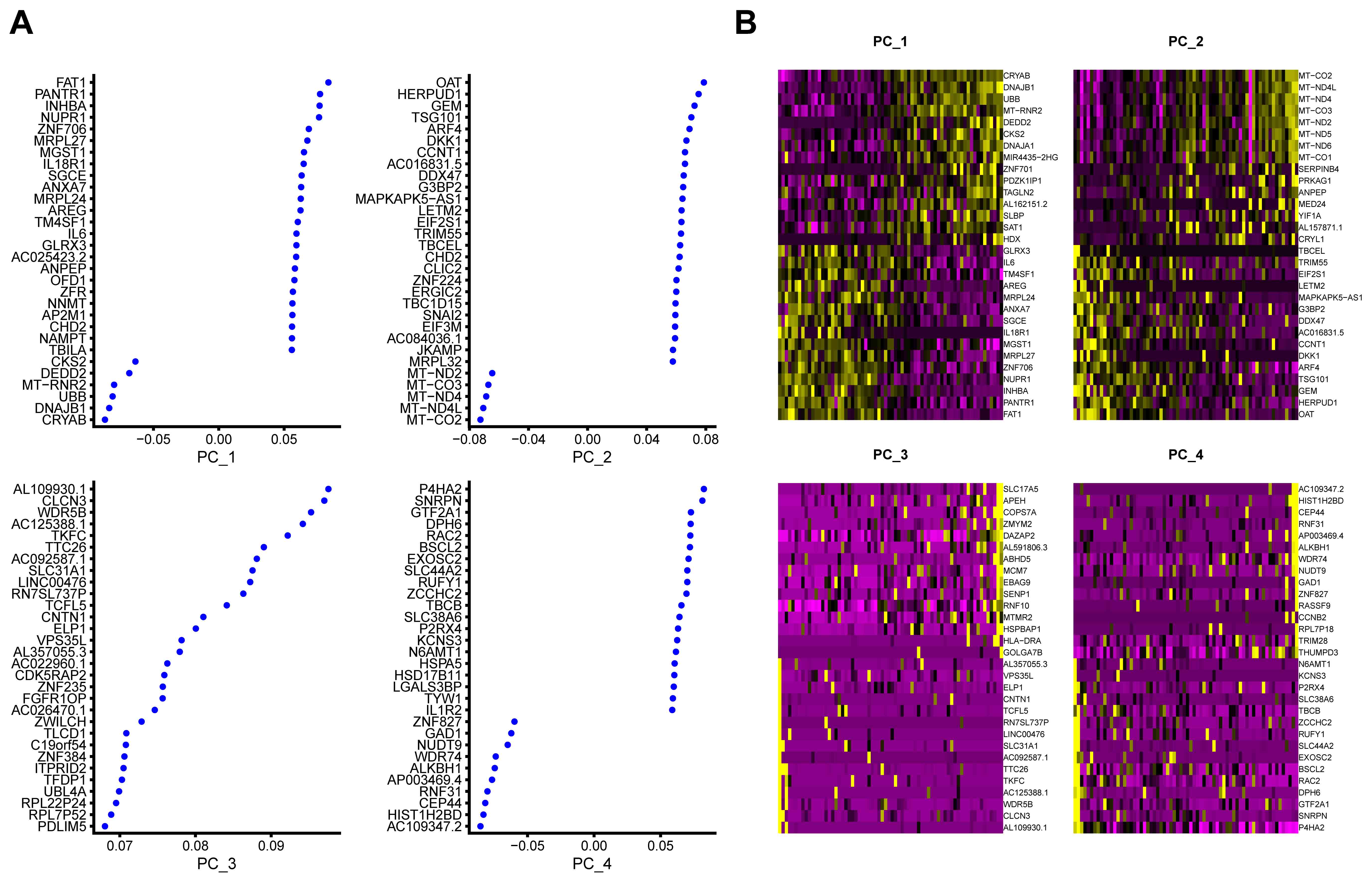

Supplement: Supplementary file 1 [file cells-12-00180-s001.zip › Supplementary Figures/FIgure S3.jpg]

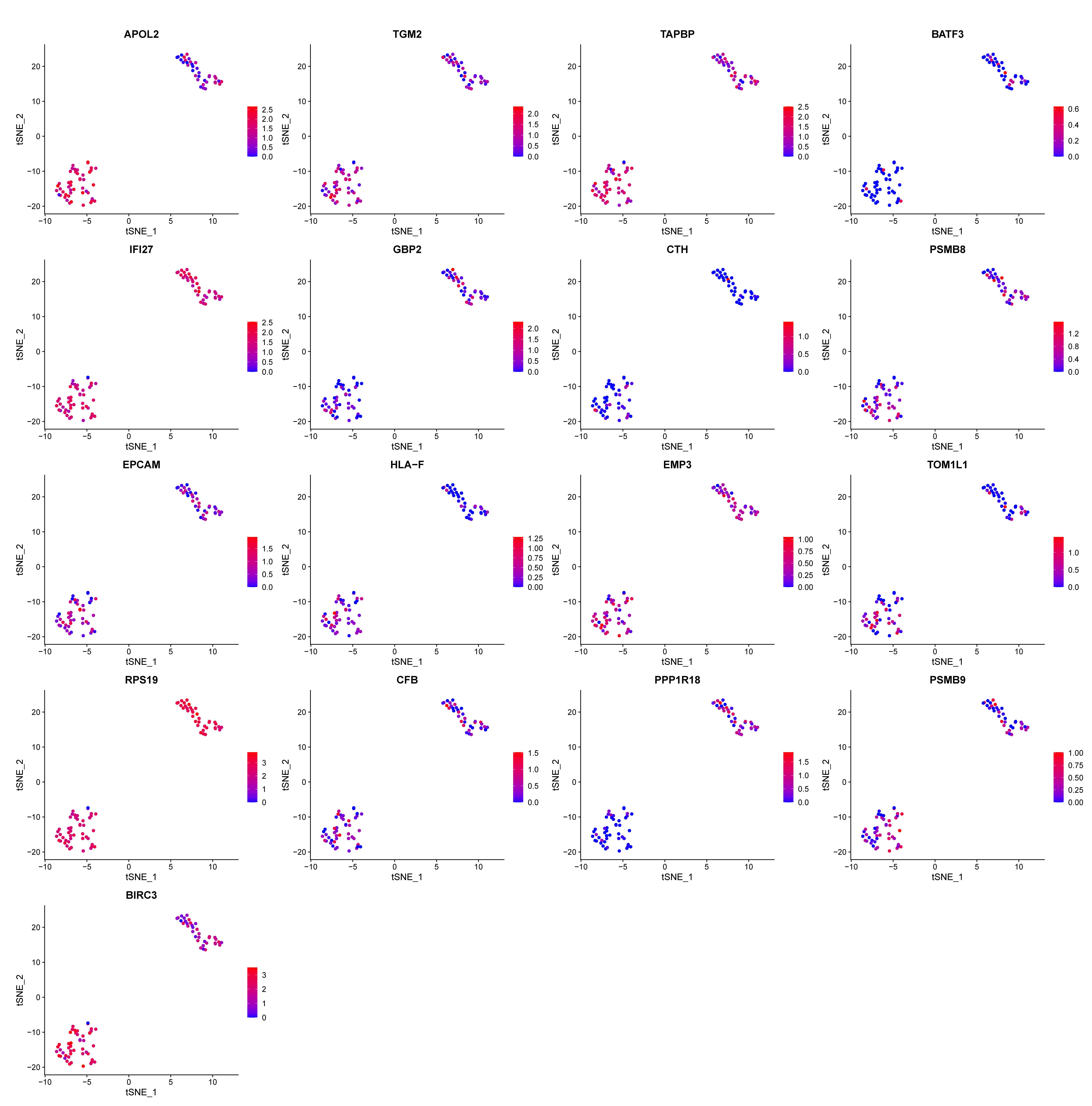

Supplement: Supplementary file 1 [file cells-12-00180-s001.zip › Supplementary Figures/FIgure S4.jpg]

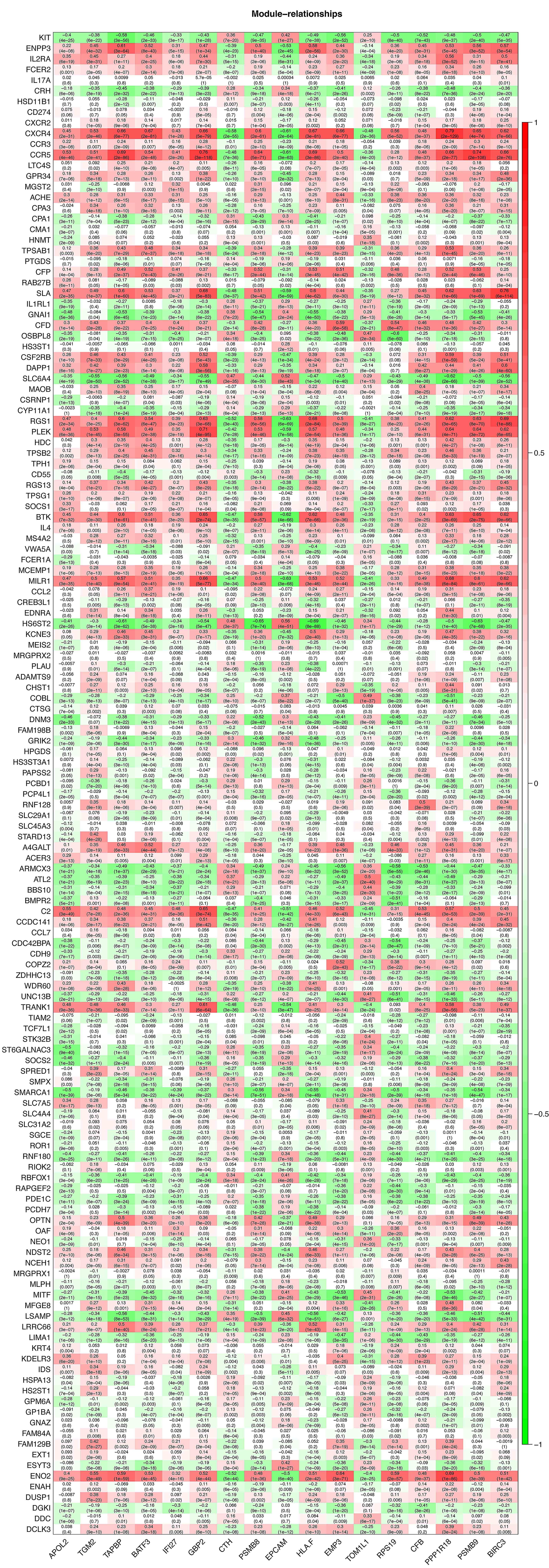

Supplement: Supplementary file 1 [file cells-12-00180-s001.zip › Supplementary Figures/Figure S5.jpg]

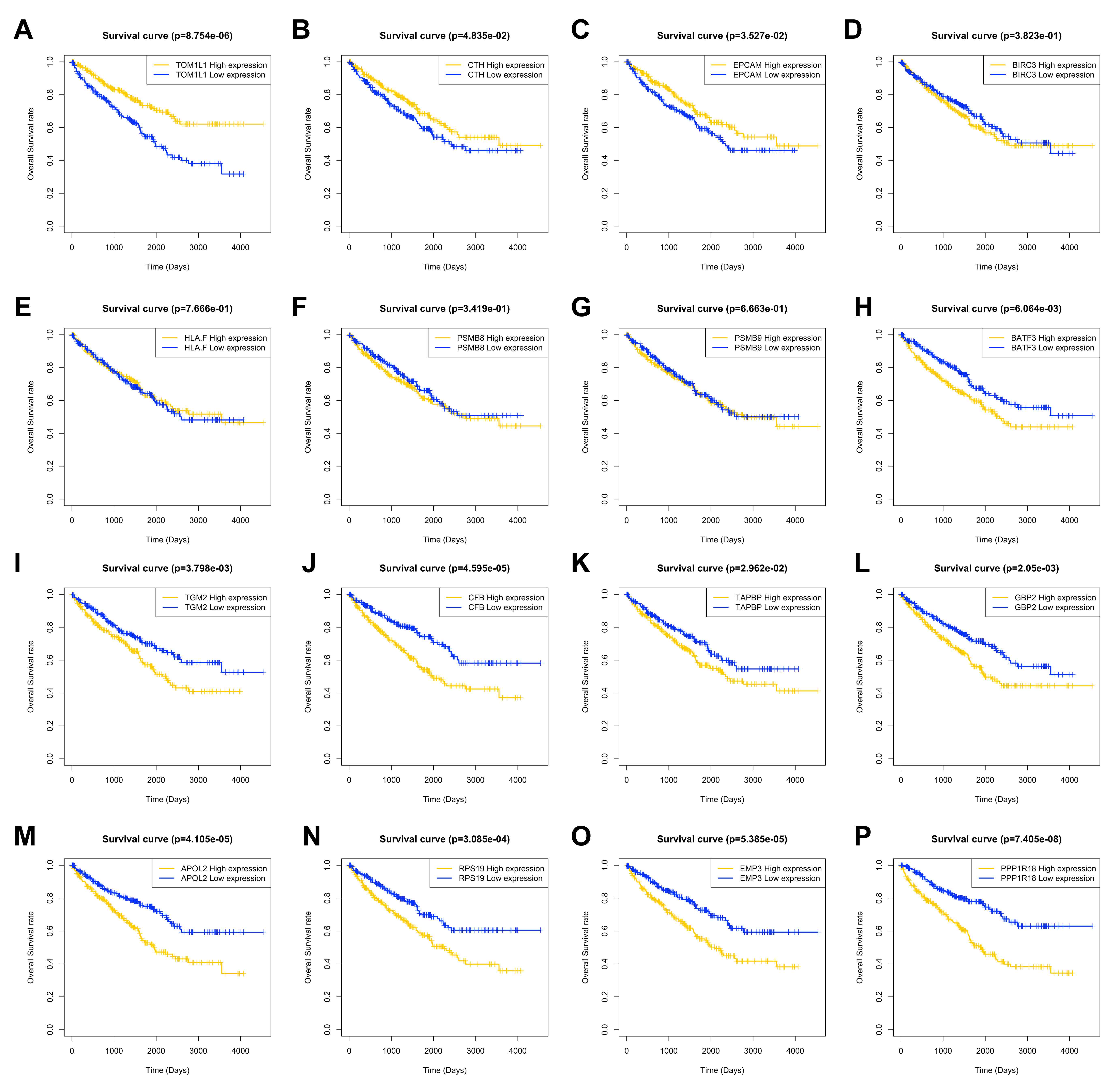

Supplement: Supplementary file 1 [file cells-12-00180-s001.zip › Supplementary Figures/FIgure S6.jpg]

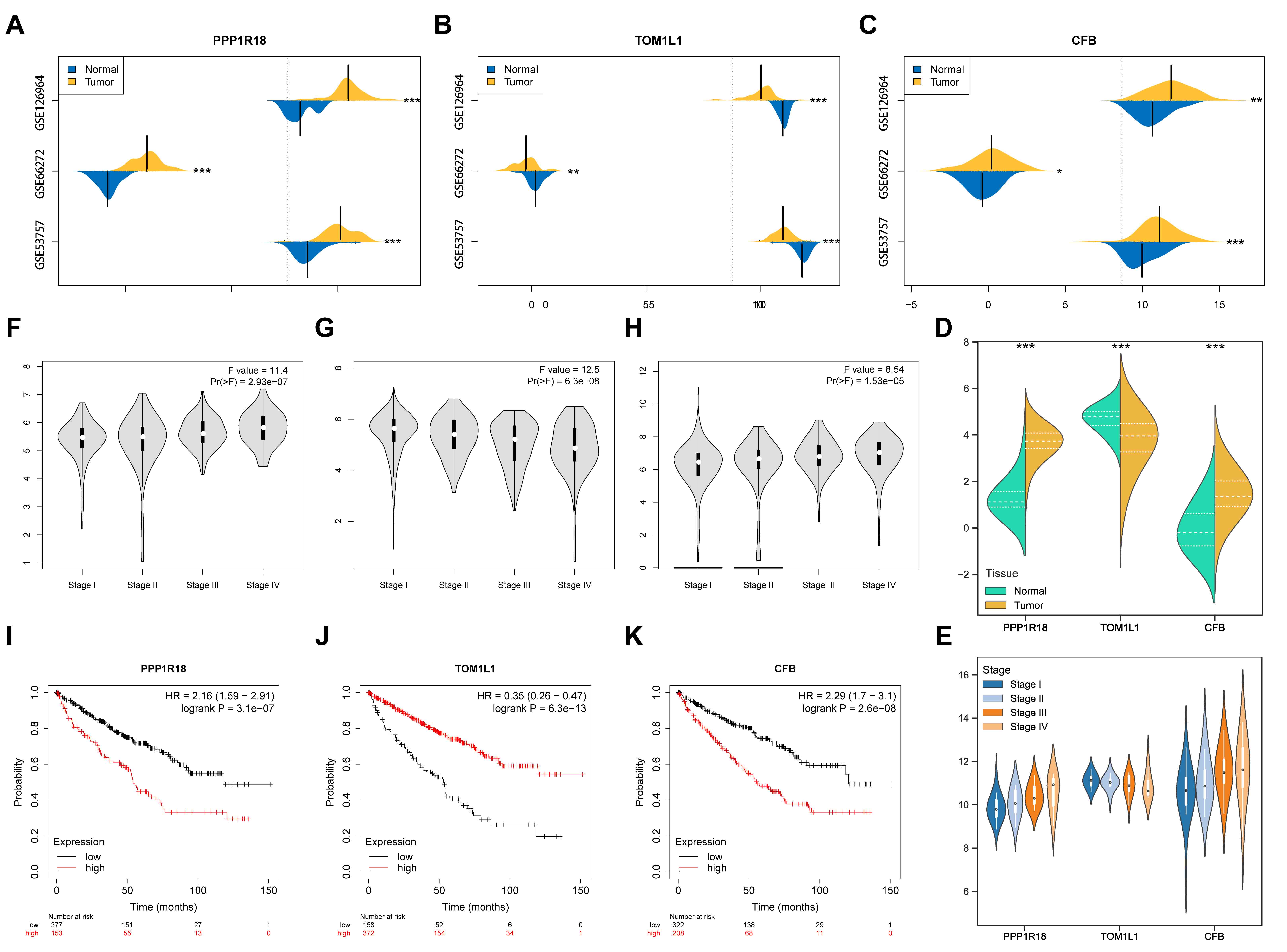

Supplement: Supplementary file 1 [file cells-12-00180-s001.zip › Supplementary Figures/FIgure S7.jpg]
